# Supplementary material for: A simple method for data partitioning based on relative evolutionary rates
Source: PeerJ. 2018 Aug 28;6:e5498. doi: 10.7717/peerj.5498 (PMC6118207; doi:10.7717/peerj.5498)
Supplement: Table S1 [file peerj-06-5498-s004.docx]

**Table S1.**

| **Missing data range** | **Arctiina** | **Calisto** | **Choreutidae** | **Coenonymphina** | **Geometridae** | **Morpho** | **Noctuidae** | **Pieridae** |
| --- | --- | --- | --- | --- | --- | --- | --- | --- |
| 0-10% | 11% | 10% | 37% | 47% | 10% | 16% | 45% | 28% |
| 10-20% | 10% | 1% | 26% | 46% | 26% | 26% | 22% | 16% |
| 20-30% | 14% | 5% | 1% | 5% | 29% | 20% | 12% | 35% |
| 30-40% | 22% | 33% | 27% | 1% | 15% | 8% | 4% | 8% |
| 40-50% | 12% | 24% | 2% | 0% | 5% | 15% | 9% | 3% |
| 50-60% | 17% | 22% | 3% | 0% | 3% | 3% | 5% | 5% |
| 60-70% | 2% | 2% | 4% | 0% | 13% | 6% | 1% | 2% |
| 70-80% | 13% | 2% | 0% | 0% | 1% | 6% | 1% | 2% |
| 80-90% | 0% | 0% | 0% | 1% | 0% | 0% | 0% | 0% |
| 90-100% | 0% | 0% | 0% | 0% | 0% | 0% | 1% | 0% |
| **Cumulative**  **missing data** | |  |  |  |  |  |  |  |
| 0-10% | 11% | 10% | 37% | 47% | 10% | 16% | 45% | 28% |
| 0-20% | 21% | 11% | 62% | 93% | 36% | 42% | 67% | 44% |
| 0-30% | 34% | 16% | 63% | 97% | 64% | 62% | 79% | 79% |
| 0-40% | 56% | 50% | 90% | 99% | 79% | 70% | 84% | 88% |
| 0-50% | 68% | 74% | 93% | 99% | 84% | 85% | 92% | 91% |
| 0-60% | 85% | 96% | 96% | 99% | 87% | 88% | 97% | 96% |
| 0-70% | 87% | 98% | 100% | 99% | 99% | 94% | 98% | 98% |
| 0-80% | 100% | 100% | 100% | 99% | 100% | 100% | 99% | 100% |
| 0-90% | 100% | 100% | 100% | 100% | 100% | 100% | 99% | 100% |
| 100% | 100% | 100% | 100% | 100% | 100% | 100% | 100% | 100% |
