# Supplementary material for: A simple method for data partitioning based on relative evolutionary rates
Source: PeerJ. 2018 Aug 28;6:e5498. doi: 10.7717/peerj.5498 (PMC6118207; doi:10.7717/peerj.5498)
Supplement: Table S2 — Gene refers to partitioning by gene fragment, Codon to partitioning by codon position, and TIG to partitioning by relative evolutionary rate as estimated with the program TIGER with different values for the d, division factor in the RatePartitions script. See Table 3 and text for more details. [file peerj-06-5498-s005.docx]

**Table S2.**

| **Partitioning strategy** | **Arctiina** | **Calisto** | **Choreutidae** | **Coenonymphina** | **Geometridae** | **Morpho** | **Noctuidae** | **Pieridae** |
| --- | --- | --- | --- | --- | --- | --- | --- | --- |
| Gene | 8 | 6 | 8 | 5 | 8 | 8 | 8 | 8 |
| GeneGr | 3 | 4 | 4 | 4 | 6 | 4 | 6 | 5 |
| Codon | 24 | 18 | 24 | 15 | 24 | 24 | 24 | 24 |
| CodonGr | 9 | 7 | 10 | 9 | 15 | 7 | 12 | 12 |
| TIG1.5 | 4 | 4 | 3 | 4 | 7 | 2 | 4 | 6 |
| TIG2.0 | 5 | 6 | 5 | 5 | 10 | 3 | 6 | 8 |
| TIG2.5 | 7 | 7 | 6 | 7 | 13 | 4 | 8 | 11 |
| TIG3.0 | 8 | 9 | 7 | 8 | 15 | 5 | 9 | 13 |
| TIG3.5 | 10 | 10 | 8 | 9 | 18 | 5 | 11 | 16 |
| TIG3.5Gr | 6 | 5 | 6 | 6 | 12 | 4 | 7 | 10 |
| TIG4.0 | 11 | 12 | 9 | 11 | 21 | 6 | 13 | 18 |
| TIG4.0Gr | 5 | 6 | 6 | 7 | 12 | 4 | 6 | 9 |
| TIG4.5 | 12 | 13 | 10 | 12 | 24 | 7 | 14 | 20 |
| TIG4.5Gr | 5 | 7 | 6 | 7 | 16 | 4 | 6 | 8 |
