# Supplementary material for: A simple method for data partitioning based on relative evolutionary rates
Source: PeerJ. 2018 Aug 28;6:e5498. doi: 10.7717/peerj.5498 (PMC6118207; doi:10.7717/peerj.5498)
Supplement: Table S3 — A BIC value is provided for each partitioning strategy for each of the eight datasets analysed. [file peerj-06-5498-s006.docx]

**Table S3.**

|  | **Arctiina** | **Calisto** | **Choreutidae** | **Coenonymphina** | **Geometridae** | **Morpho** | **Noctuidae** | **Pieridae** |
| --- | --- | --- | --- | --- | --- | --- | --- | --- |
| Gene | 103588.3 | 87177.4 | 121293.6 | 128442.5 | 382534.8 | 59014.3 | 205415.1 | 276035.7 |
| GeneGr | 103354.5 | 87099.6 | 121177.7 | 128404.7 | 382496.7 | 58800.9 | 205319.5 | 275916.7 |
| Codon | 99273.9 | 83748.5 | 115115.5 | 123829.3 | 376164.4 | 55469.3 | 198365.6 | 267225.6 |
| CodonGr | 98673.3 | 83428.7 | 114576.1 | 123580.8 | 375850.6 | 54927.7 | 197818.6 | 266681.7 |
| TIG1.5 | 101260.8 | 84437.8 | 116733.6 | 125588.2 | 375810.5 | 57182.9 | 201759.7 | 269343.1 |
| TIG2.0 | 99843.4 | 83579.5 | 114543.8 | 124119.5 | 374256.1 | 55097.7 | 199902.7 | 268516.3 |
| TIG2.5 | 99139.5 | 82675.5 | 113601.1 | 123523.9 | 373629.2 | 53915.3 | 198886.4 | 267750.6 |
| TIG3.0 | 98319.7 | 82420.7 | 112878.2 | 122718.0 | 372951.4 | 53384.2 | 198127.0 | 267597.3 |
| TIG3.5 | 98040.4 | 82178.5 | 112689.4 | 122695.1 | 372716.9 | 52049.1 | 197833.5 | 267194.0 |
| TIG3.5Gr | 97827.8 | 81951.5 | 112589.6 | 122586.0 | 372539.6 | 52037.1 | 197625.7 | 266886.8 |
| TIG4.0 | 97912.5 | 82009.3 | 112490.3 | 122742.6 | 372251.8 | 51988.2 | 198011.7 | 266948.9 |
| TIG4.0Gr | **97627.6** | 81719.1 | 112394.9 | **122551.9** | **372020.7** | **51923.3** | 197614.8 | 266550.7 |
| TIG4.5 | 97994.4 | 81985.0 | 112464.7 | 122829.1 | 372480.9 | 52039.5 | 197900.9 | 267057.4 |
| TIG4.5Gr | 97664.9 | **81666.5** | **112322.2** | 122614.8 | 372190.9 | 51949.7 | **197584.6** | **266541.1** |
