# Supplementary material for: A simple method for data partitioning based on relative evolutionary rates
Source: PeerJ. 2018 Aug 28;6:e5498. doi: 10.7717/peerj.5498 (PMC6118207; doi:10.7717/peerj.5498)
Supplement: Table S4 — The number of internal branches for a fully resolved tree from each dataset is stated together with the percentage of internal branches that are different between the analyses of datasets from the two different partitioning strategies. [file peerj-06-5498-s007.docx]

**Table S4.**

| **Dataset** | **RFD**  **value** | **No. internal branches** | **% internal branches different** |
| --- | --- | --- | --- |
| Choreutidae | 2 | 38 | 5% |
| Pieridae | 6 | 107 | 6% |
| *Morpho* | 4 | 28 | 14% |
| Arctiina | 22 | 110 | 20% |
| *Calisto* | 20 | 87 | 23% |
| Noctuidae | 18 | 75 | 24% |
| Coenonymphina | 16 | 66 | 24% |
| Geometridae | 58 | 161 | 36% |
