# Supplementary material for: A simple method for data partitioning based on relative evolutionary rates
Source: PeerJ. 2018 Aug 28;6:e5498. doi: 10.7717/peerj.5498 (PMC6118207; doi:10.7717/peerj.5498)
Supplement: Figure S1 — In each panel the tree resulting from the gene and codon partitioned analyses is shown on the left and the one from the TIGER partitioned analysis on the right: Arctiina (a), Calisto (b), Choreutidae (c), Coenonymphina (d), Geometridae (e), Morpho (f), Noctuidae (g), and Pieridae (h). [file peerj-06-5498-s008.pdf]

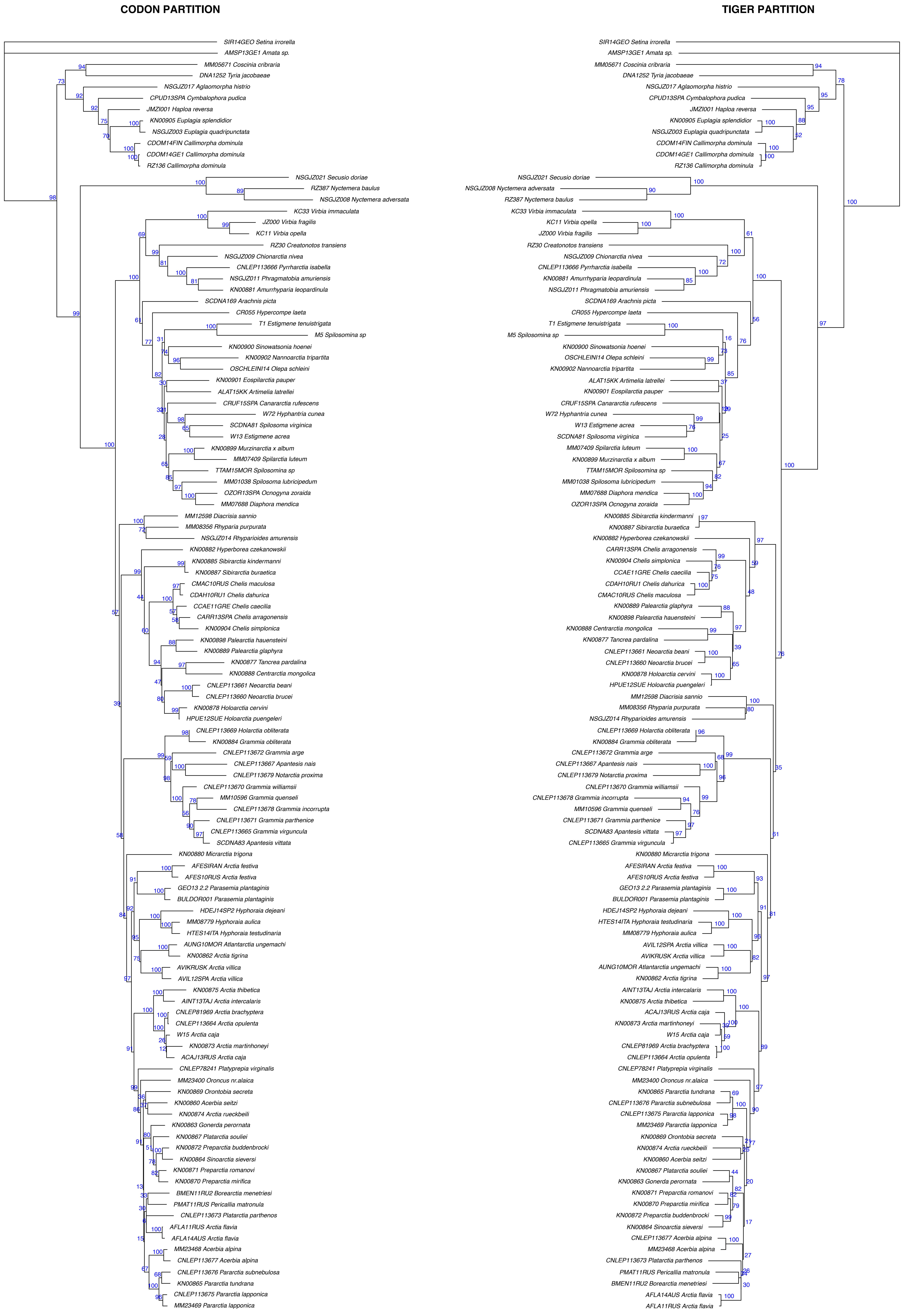

Figure S1a. Arctiina

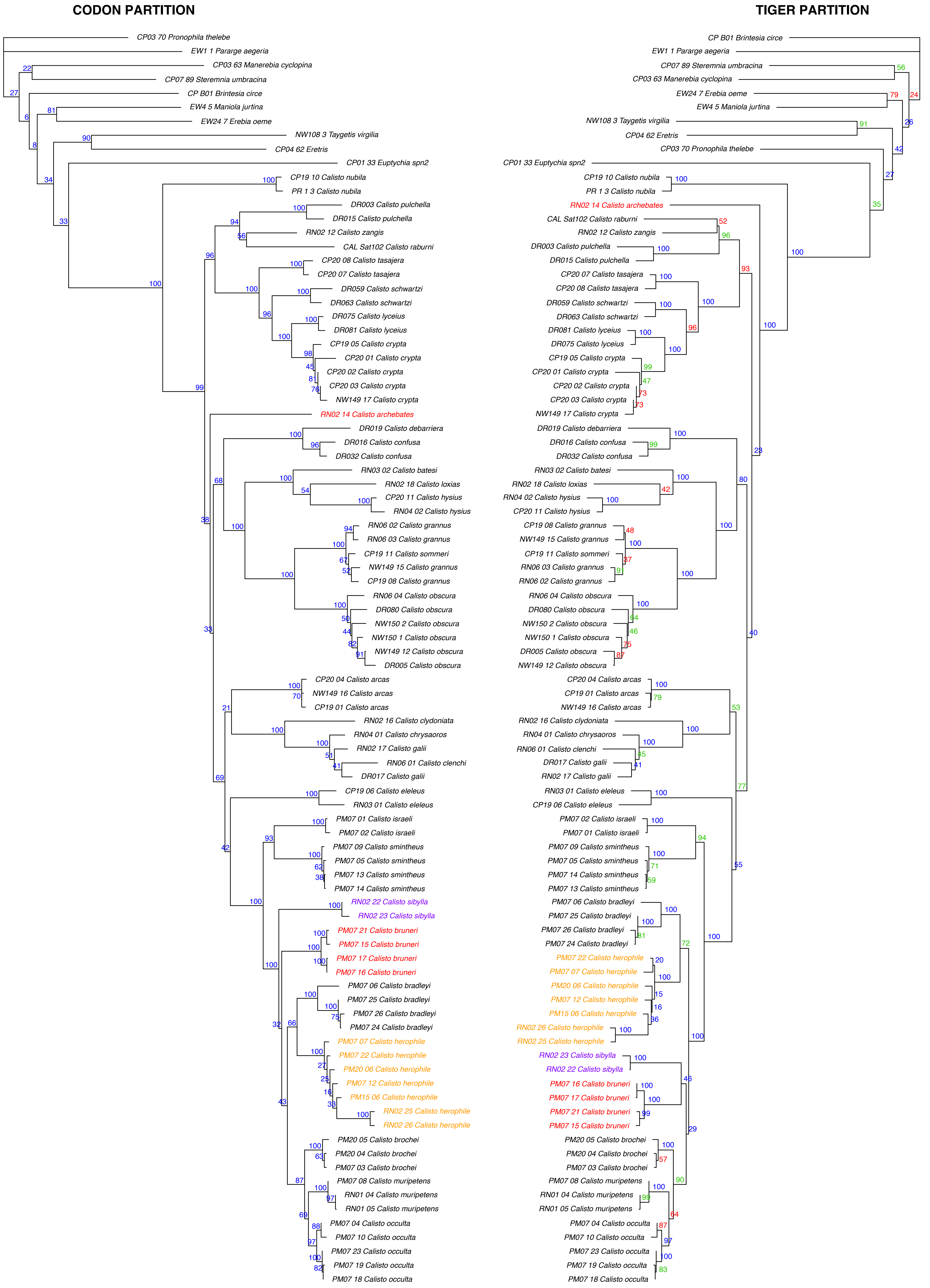

Figure S1b. ***Calisto***

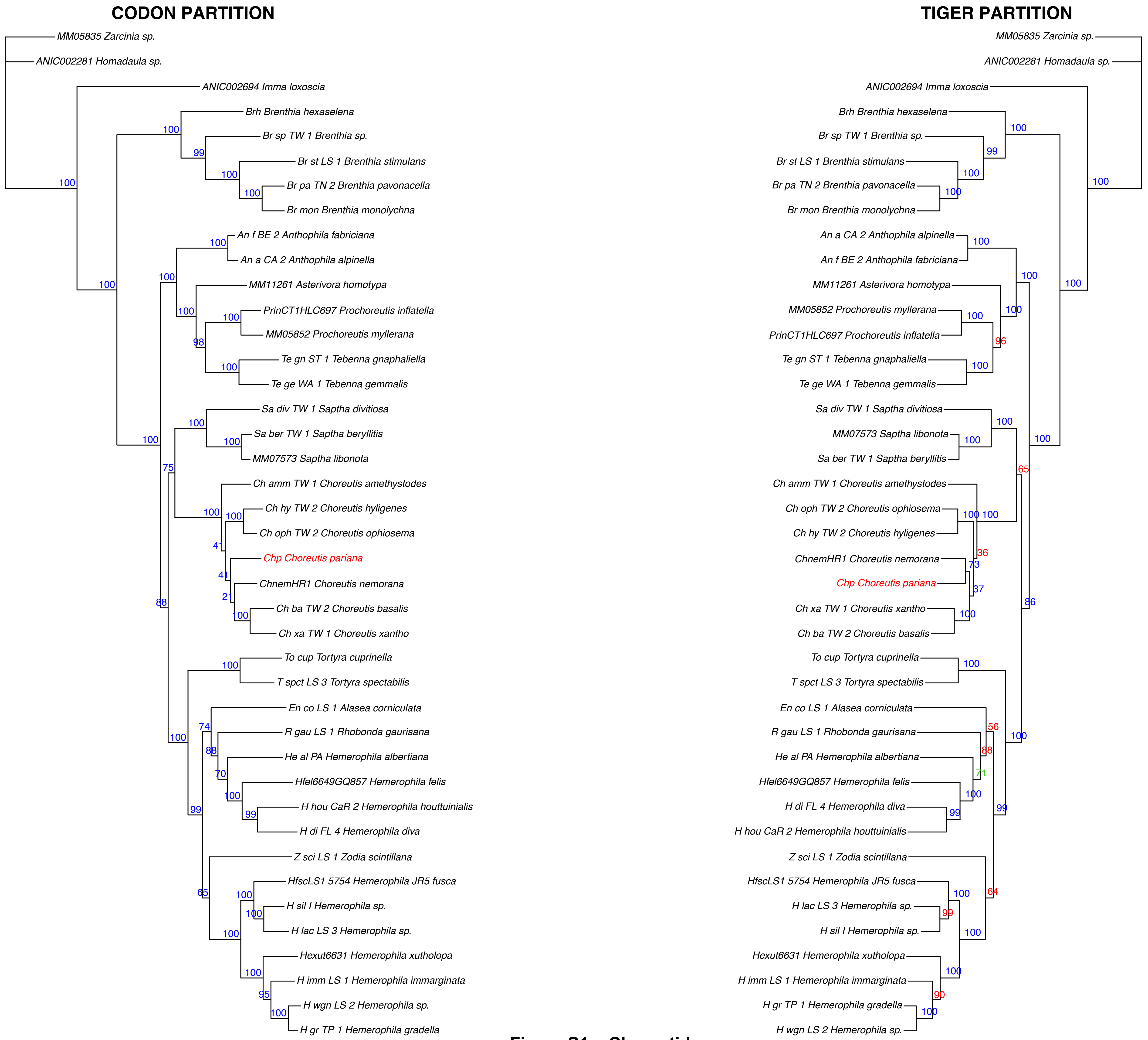

Figure S1c. Choreutidae

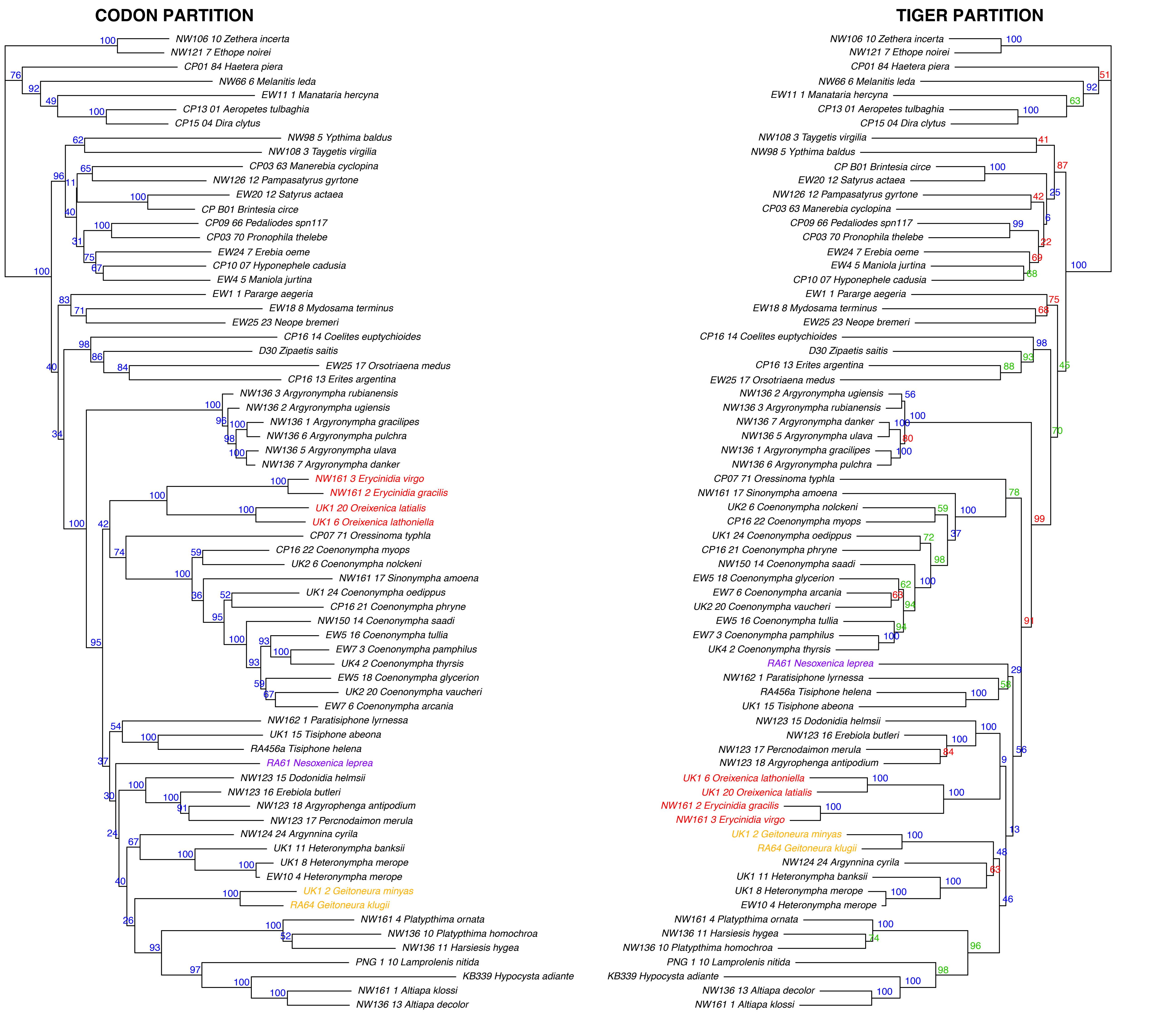

Figure S1d. Coenonymphina

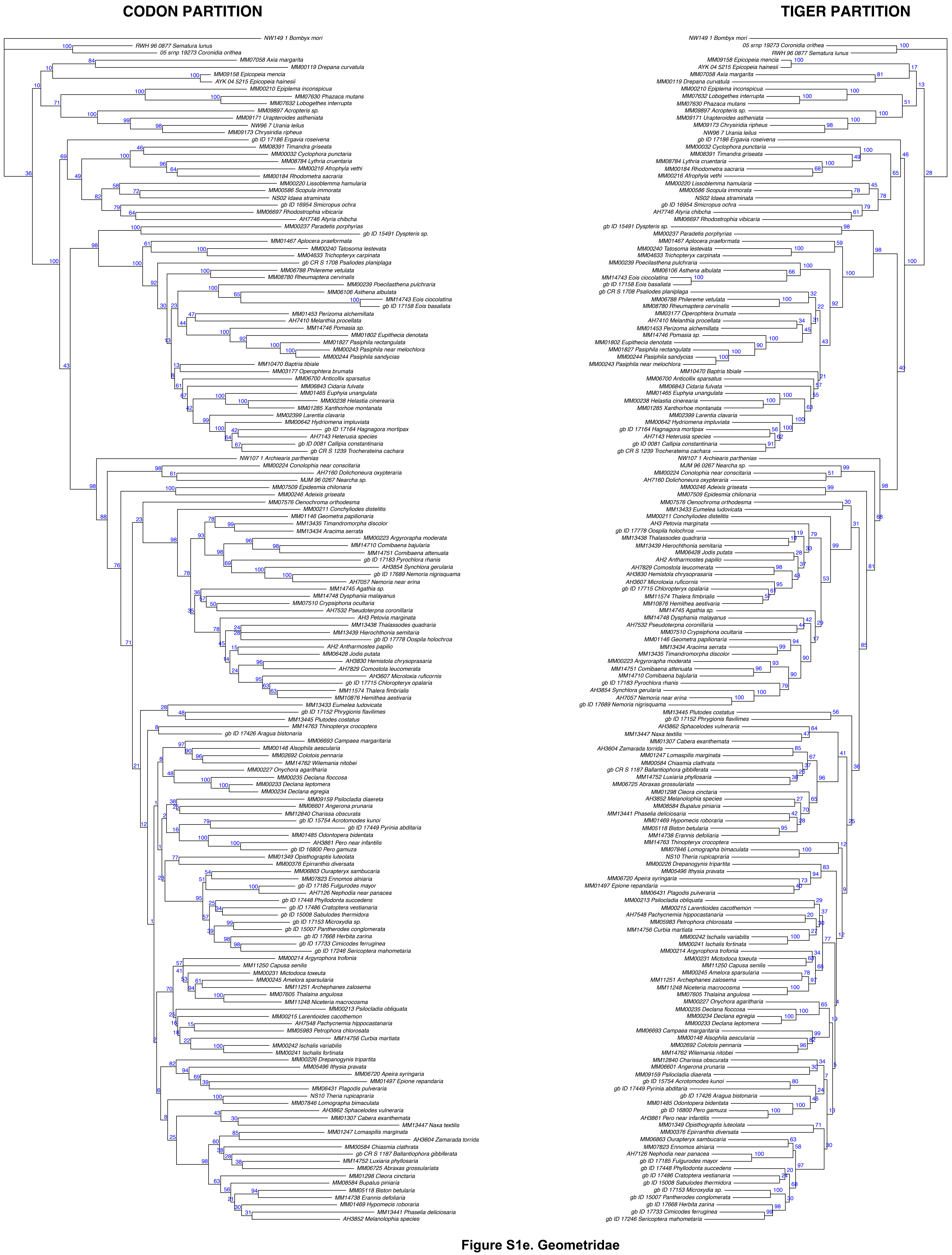

Figure S1e. Geometridae

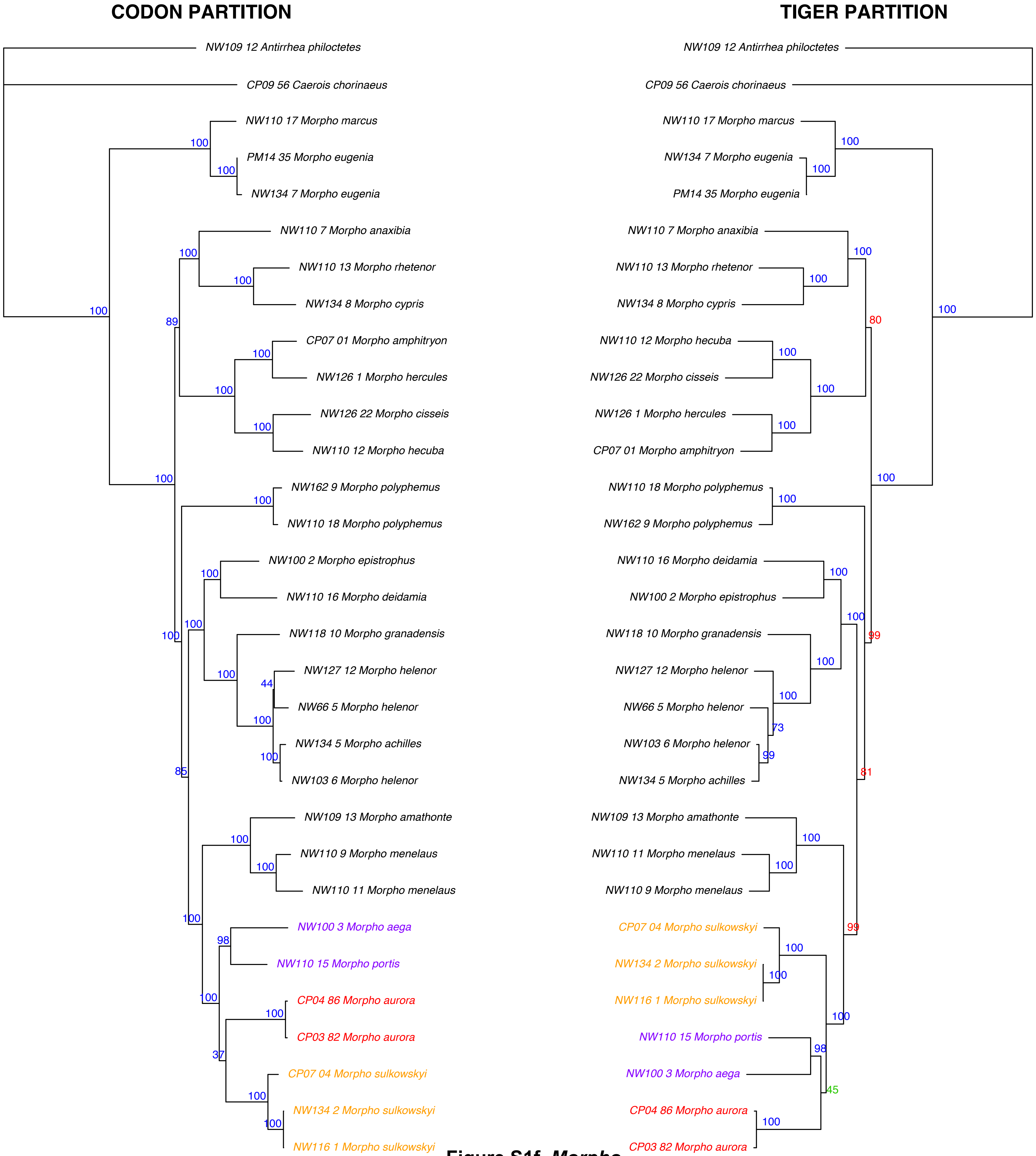

Figure S1f. *Morpho*

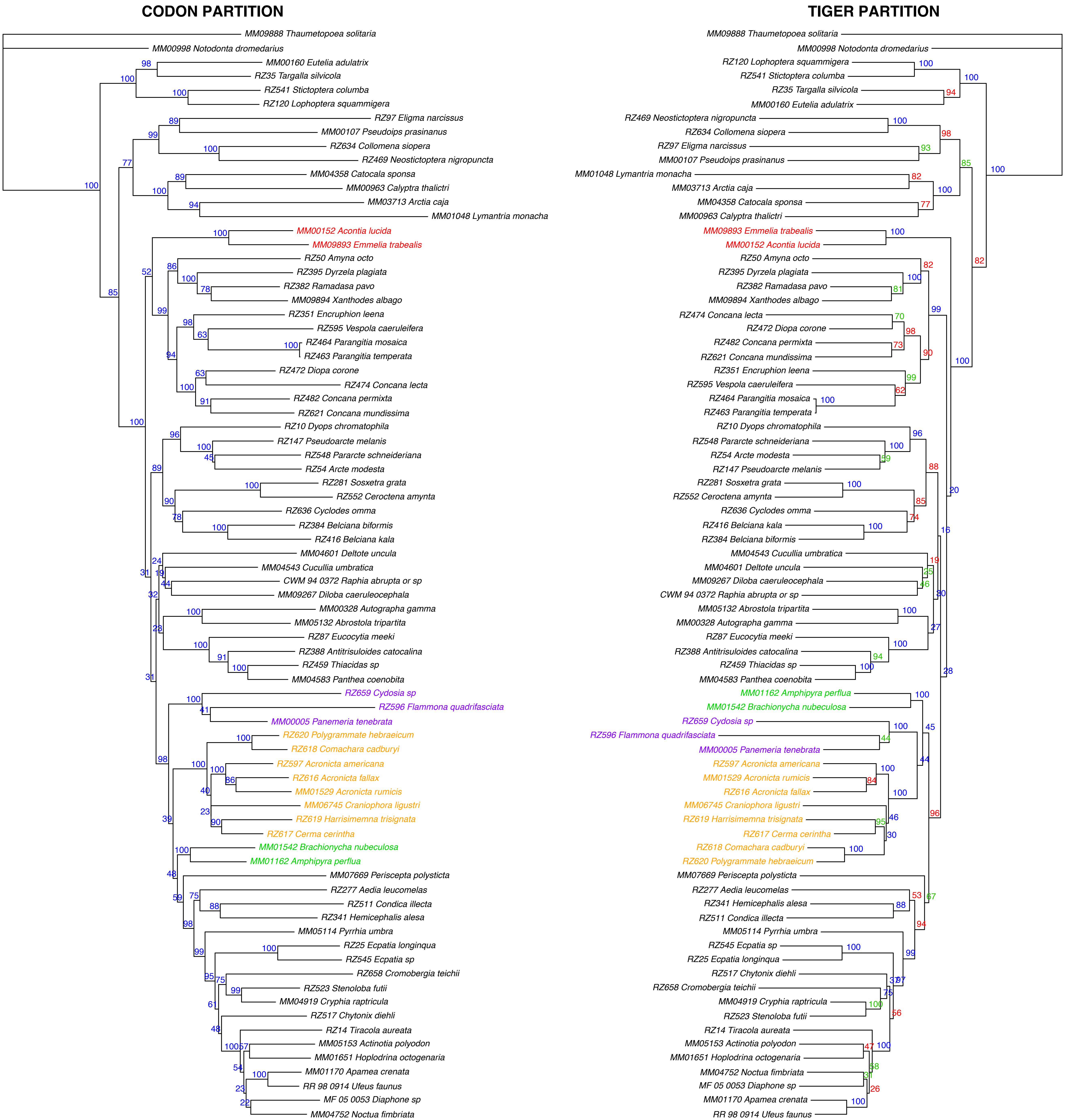

Figure S1g. Noctuidae

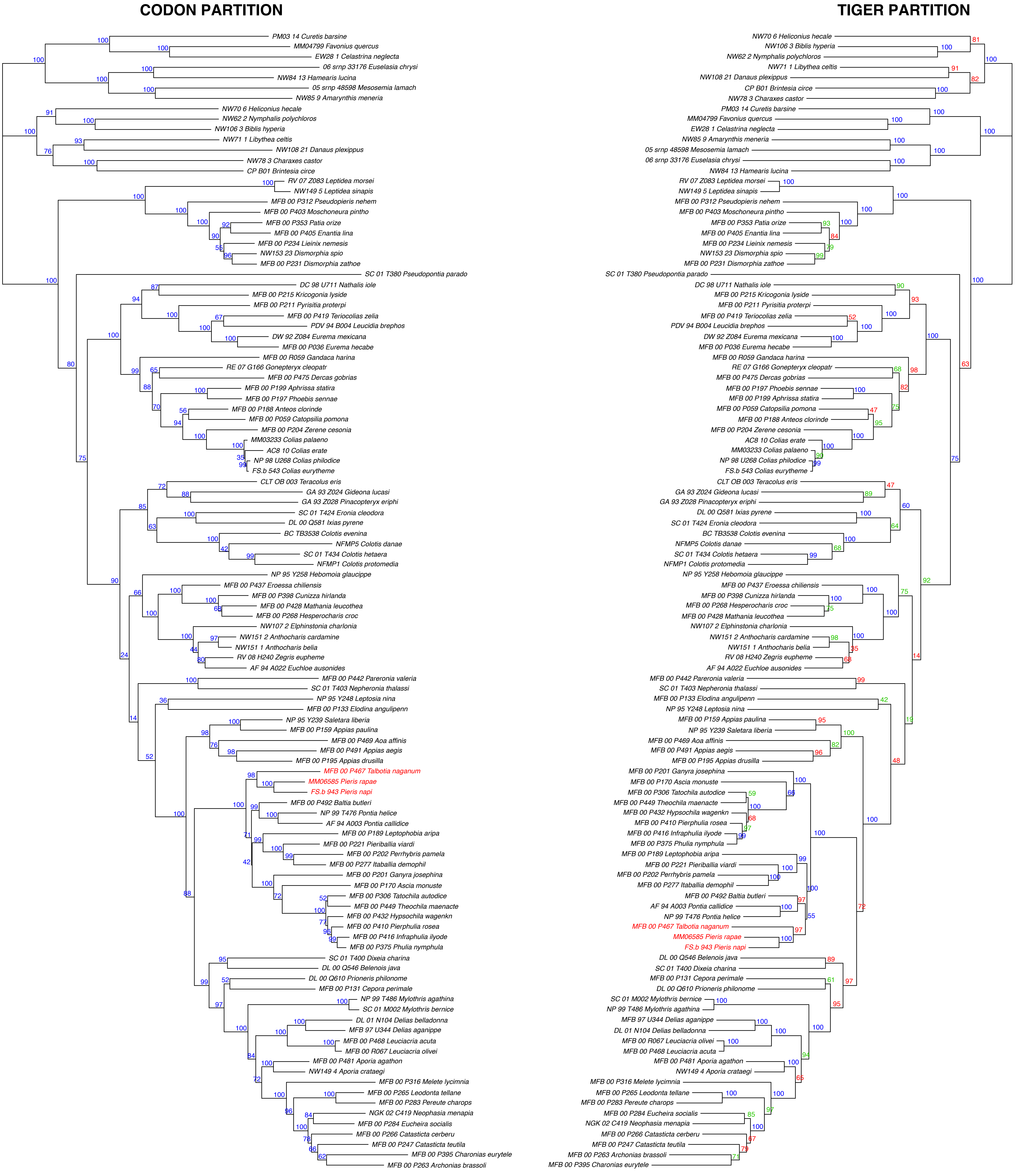

Figure S1h. Pieridae
