# Supplementary material for: A simple method for data partitioning based on relative evolutionary rates
Source: PeerJ. 2018 Aug 28;6:e5498. doi: 10.7717/peerj.5498 (PMC6118207; doi:10.7717/peerj.5498)
Supplement: Figure S2 — Plots showing the assigned TIGER relative evolutionary rates for codon positions of each of the gene fragments analysed: Arctiina (a), Calisto (b), Choreutidae (c), Coenonymphina (d), Geometridae (e), Morpho (f), and Pieridae (g). [file peerj-06-5498-s009.pdf]

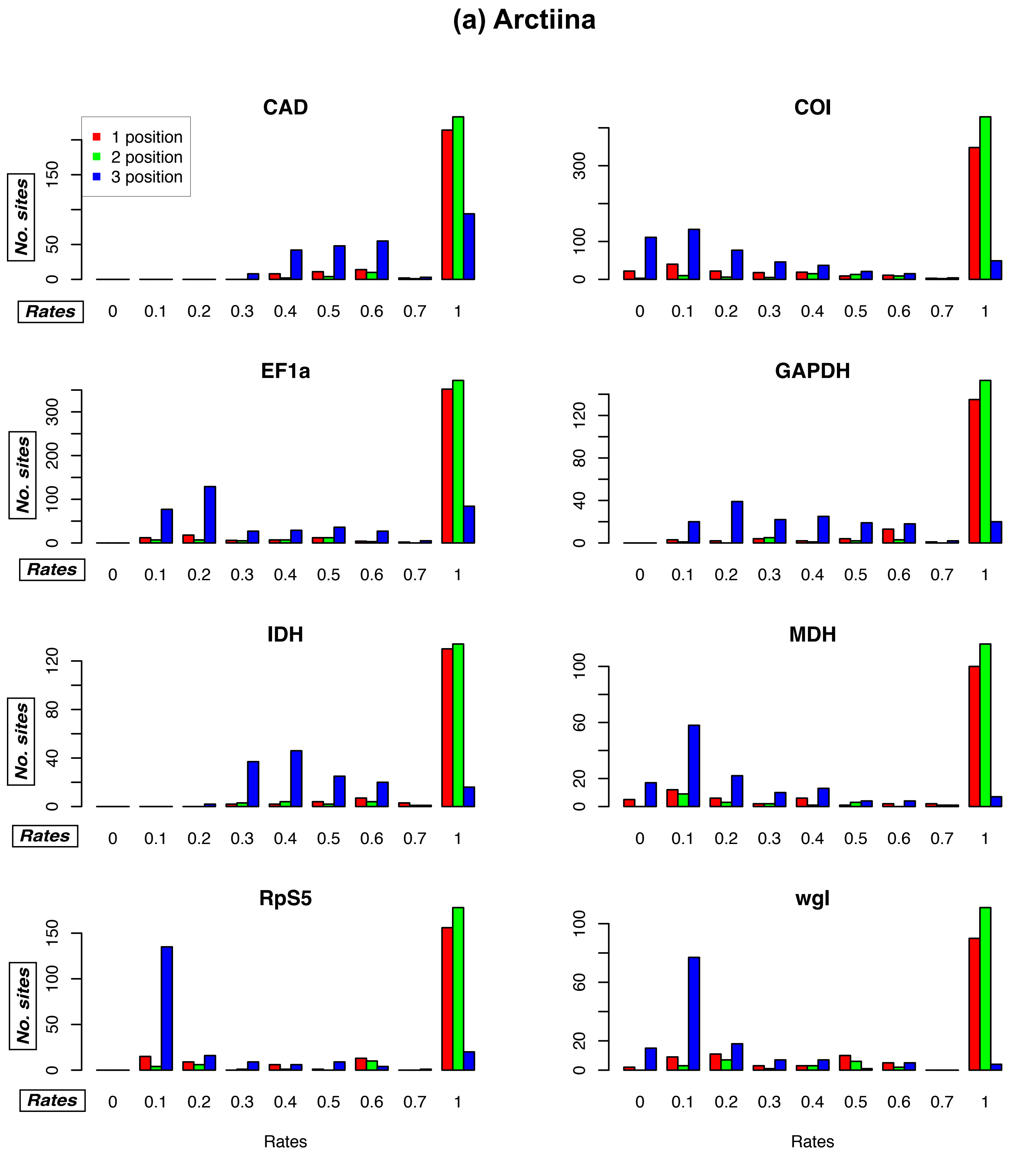

Figure S1. Plots showing the assigned TIGER rates to codon positions of each of the genes in datasets Arctiina (a), *Calisto* (b), Choreutidae (c), Coenonymphina (d), Geometridae (e), *Morpho* (f), and Pieridae (g).

(b) *Calisto*

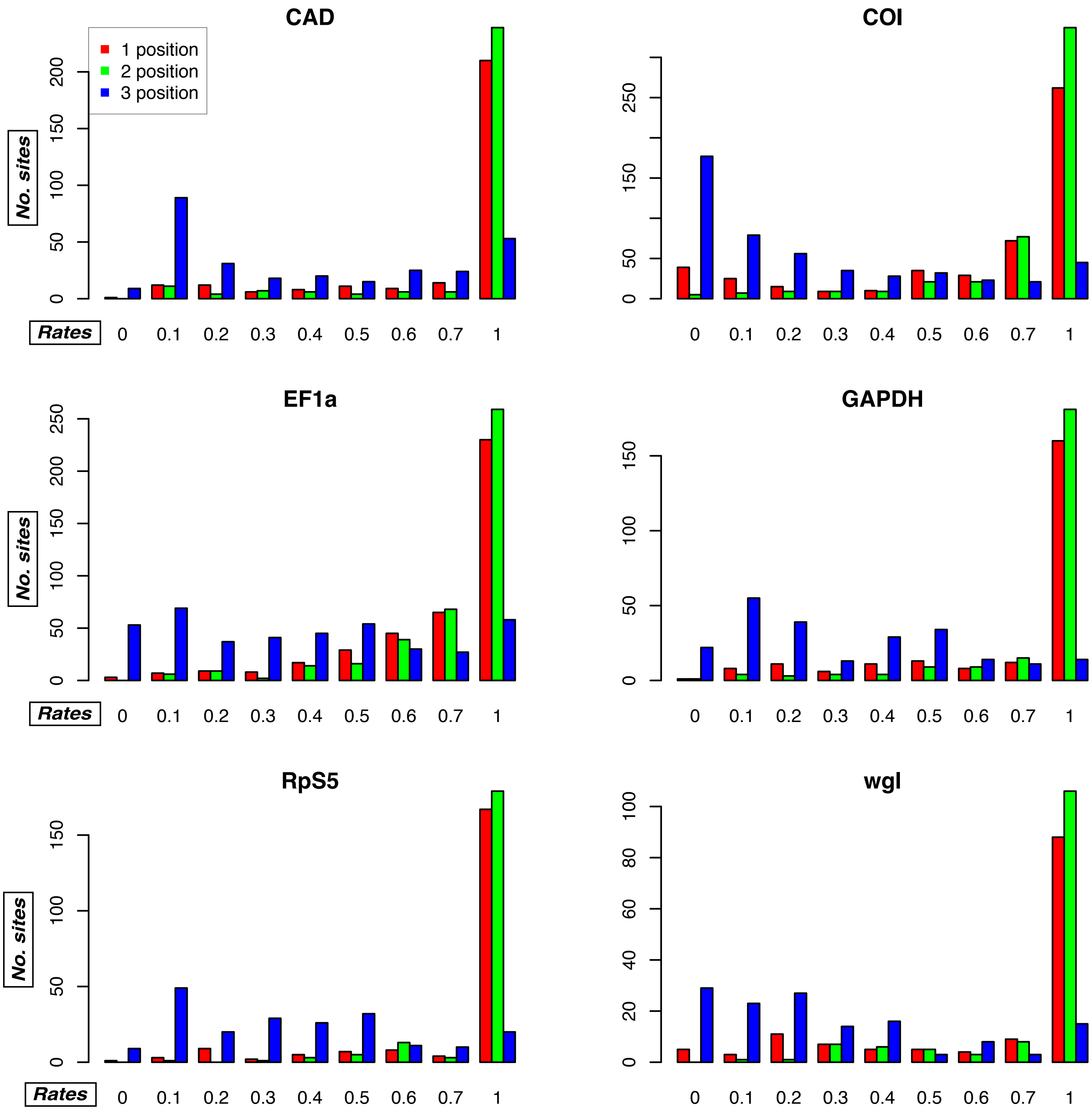

CAD
COI
EF1a
GAPDH
RpS5
wgl
1 position
2 position
3 position
No. sites
Rates
0
0.1
0.2
0.3
0.4
0.5
0.6
0.7
1

(c) Choreutidae

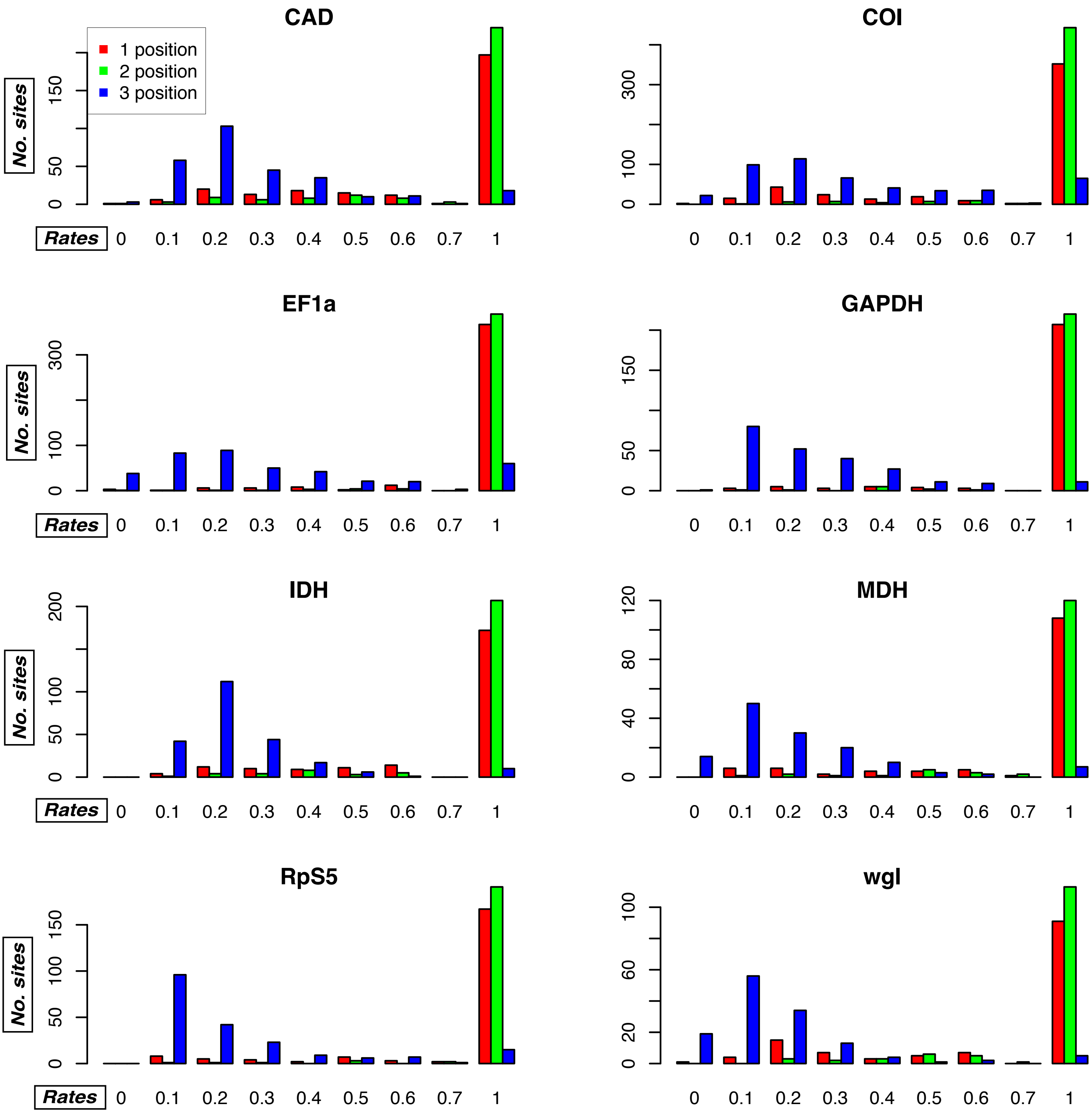

(d) Coenonymphina

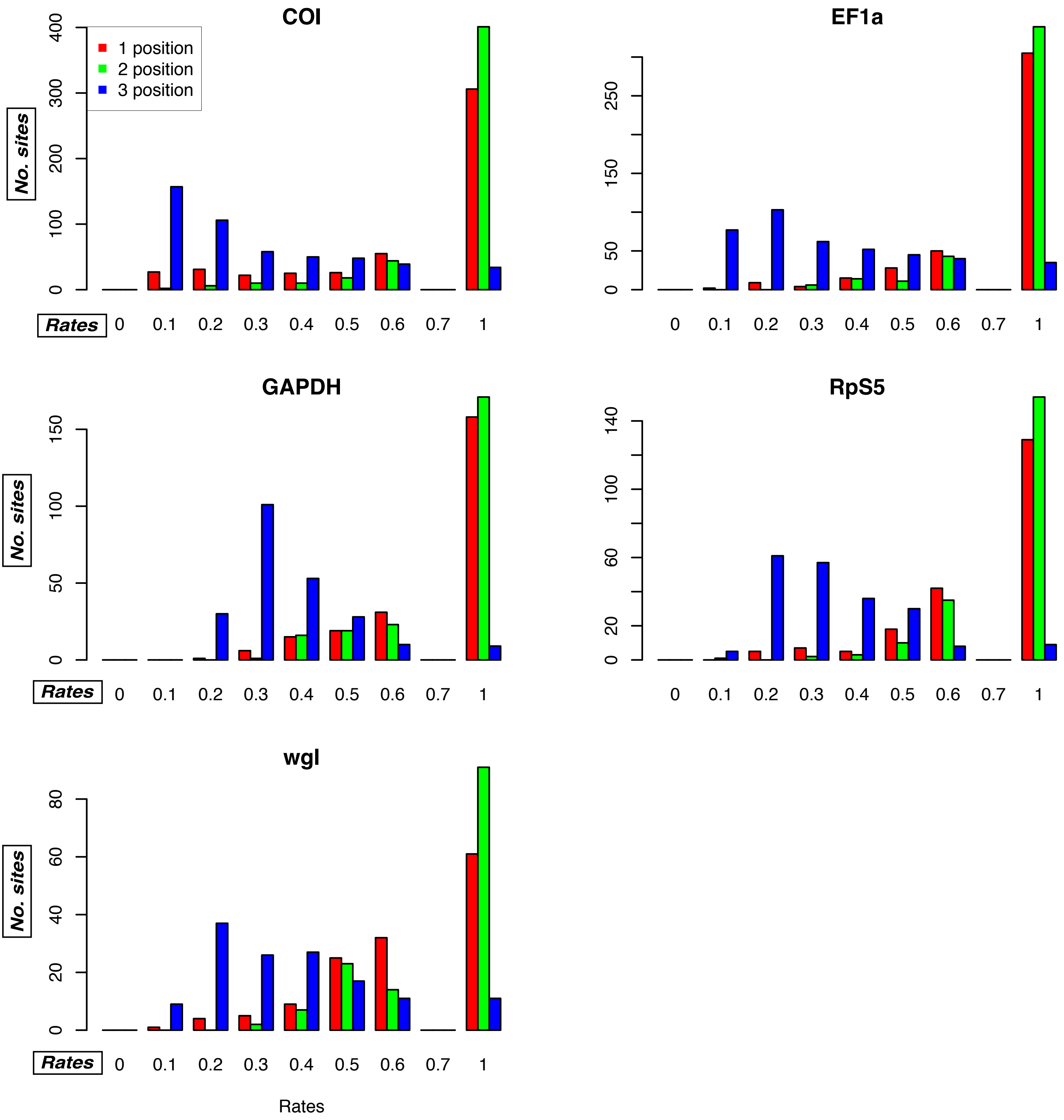

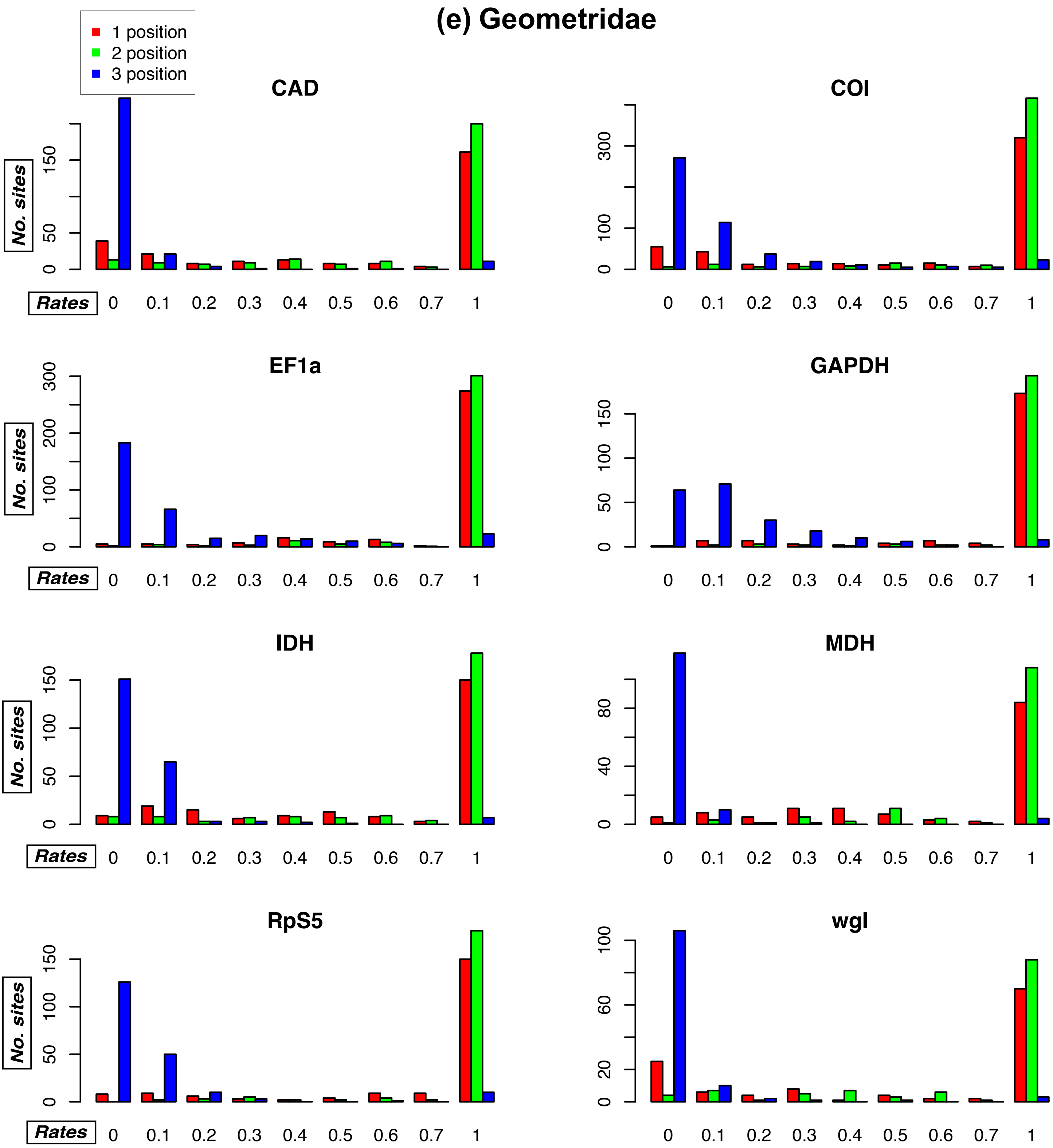

(e) Geometridae
1 position
2 position
3 position
CAD
COI
EF1a
GAPDH
IDH
MDH
RpS5
wgl
No. sites
Rates
0
0.1
0.2
0.3
0.4
0.5
0.6
0.7
1

(f) *Morpho*

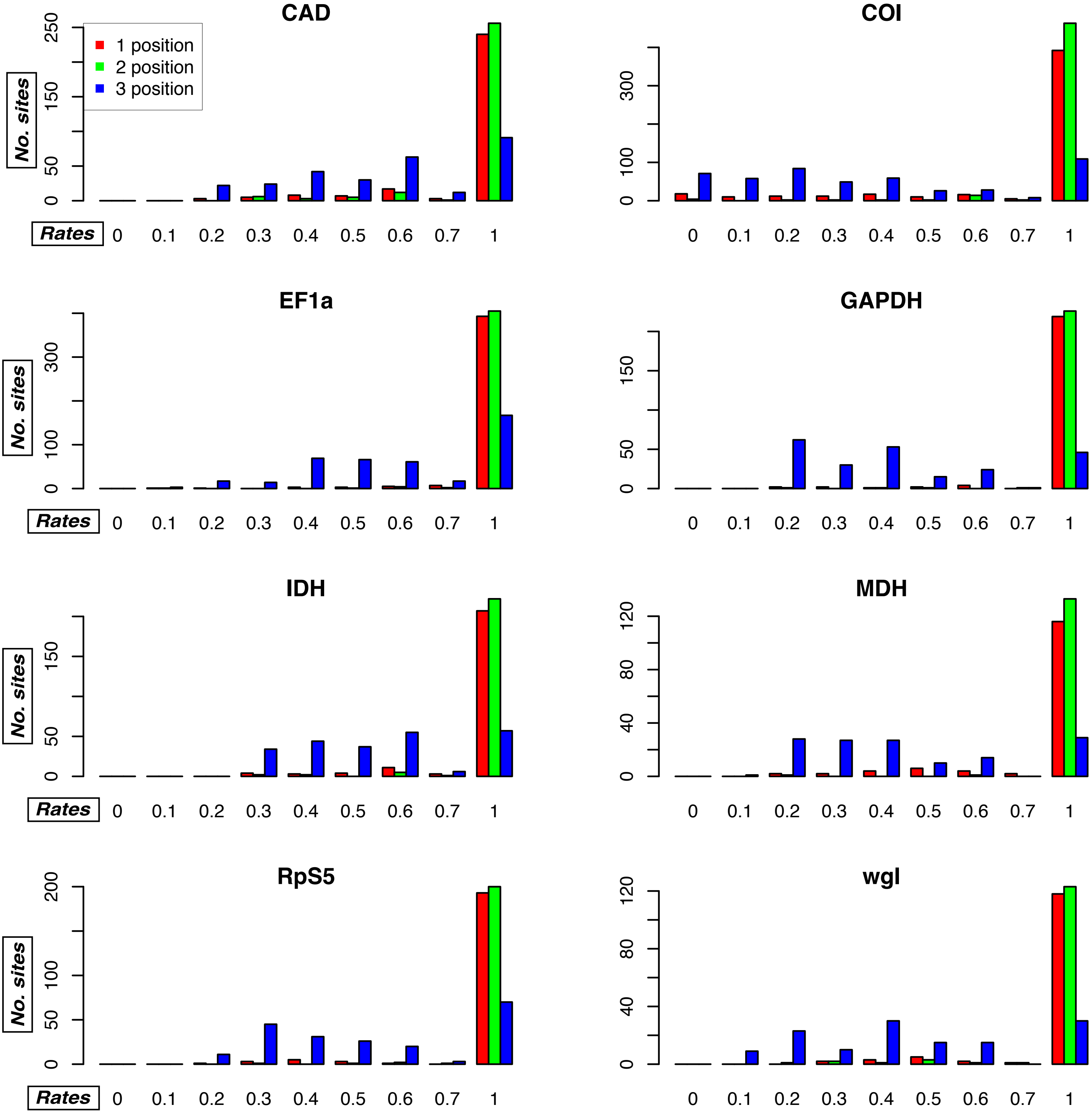
CAD
COI
EF1a
GAPDH
IDH
MDH
RpS5
wgl
1 position
2 position
3 position
No. sites
Rates
0
0.1
0.2
0.3
0.4
0.5
0.6
0.7
1

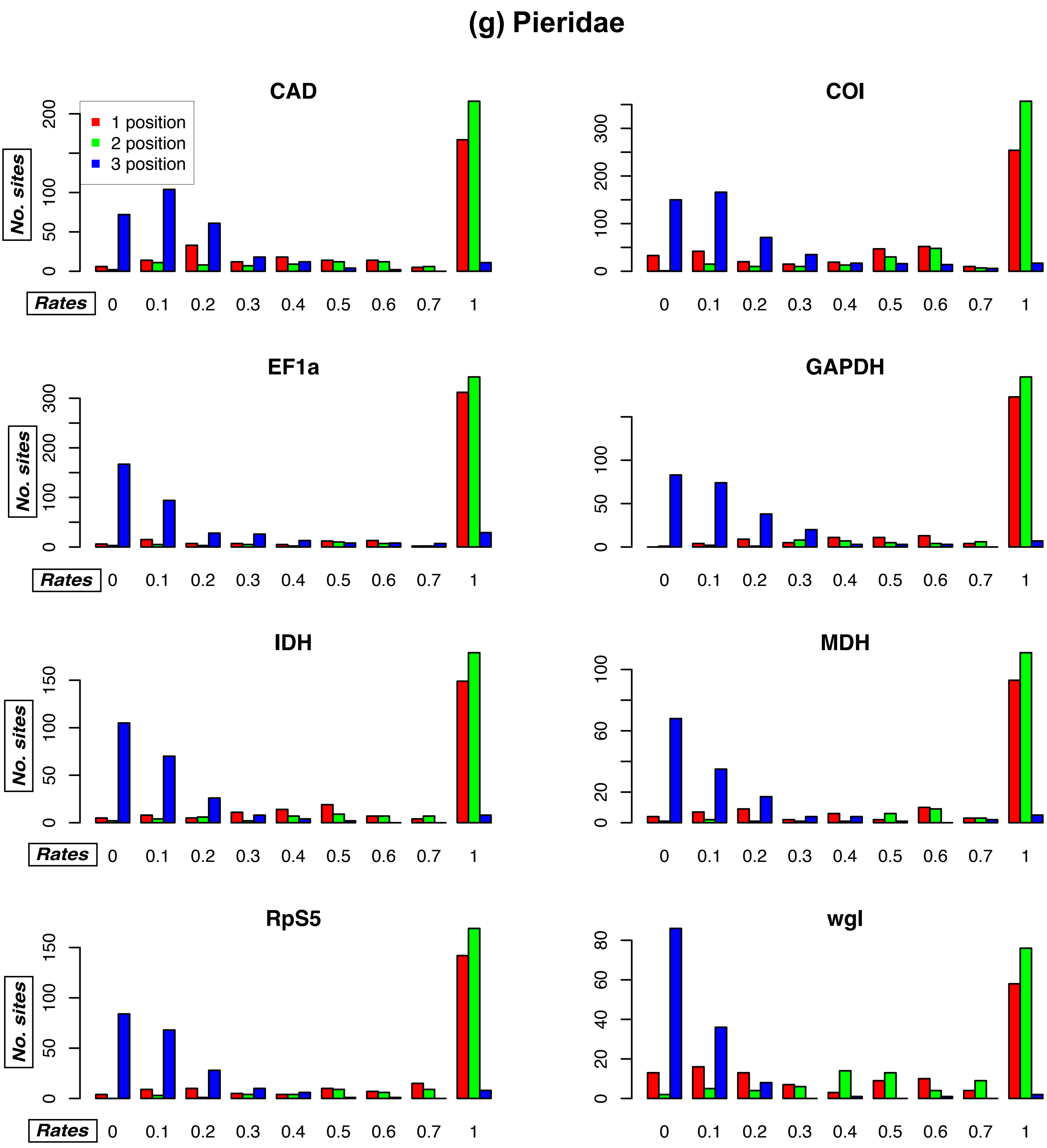

(g) Pieridae
CAD
COI
EF1a
GAPDH
IDH
MDH
RpS5
wgl
1 position
2 position
3 position
No. sites
Rates
0
0.1
0.2
0.3
0.4
0.5
0.6
0.7
1
