# Supplementary figures and images for: A simple method for data partitioning based on relative evolutionary rates
Source: PeerJ. 2018 Aug 28;6:e5498. doi: 10.7717/peerj.5498 (PMC6118207; doi:10.7717/peerj.5498)

MEIKLEJOHN ET AL PARTITION

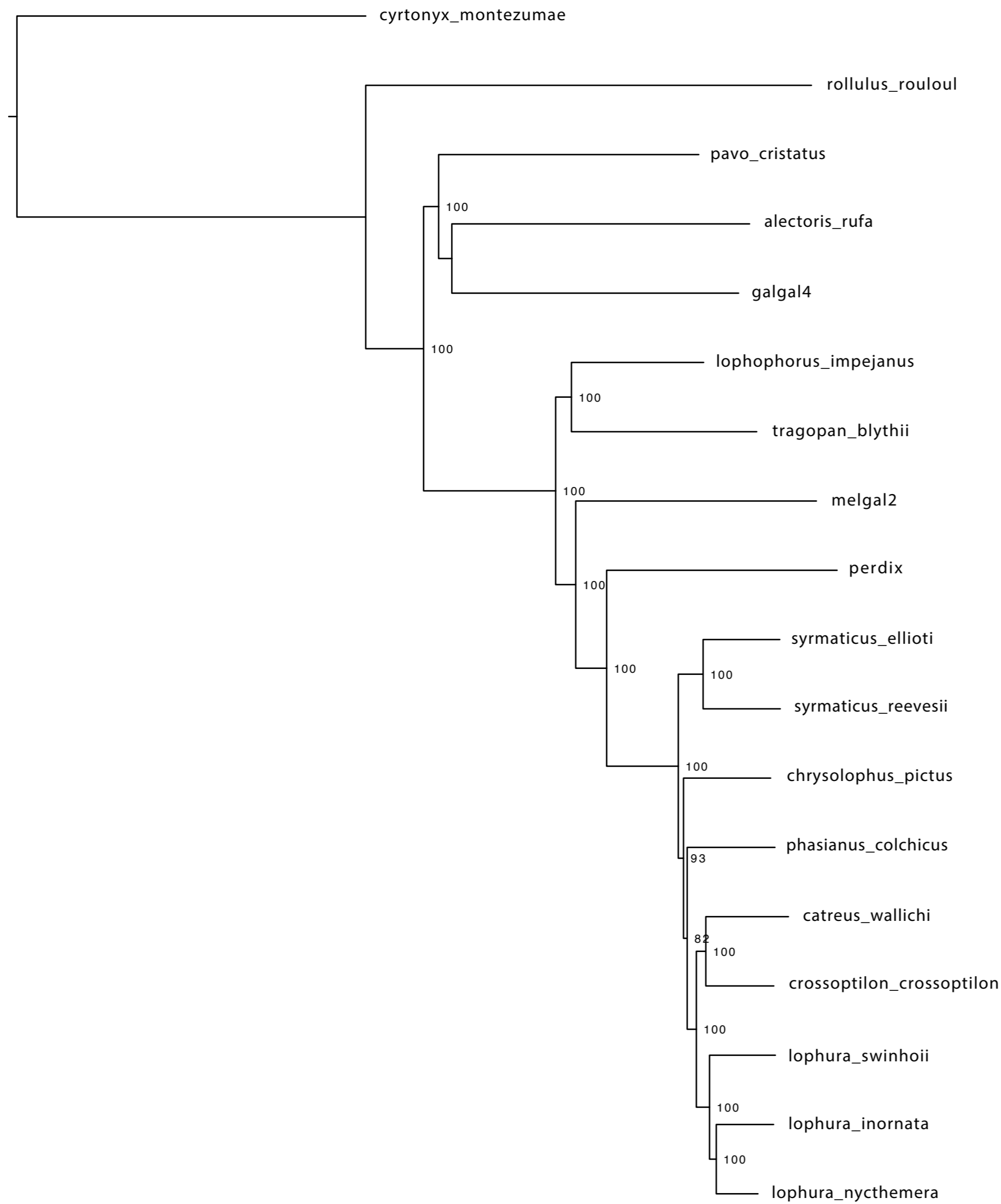

0.0030

TIGER PARTITION

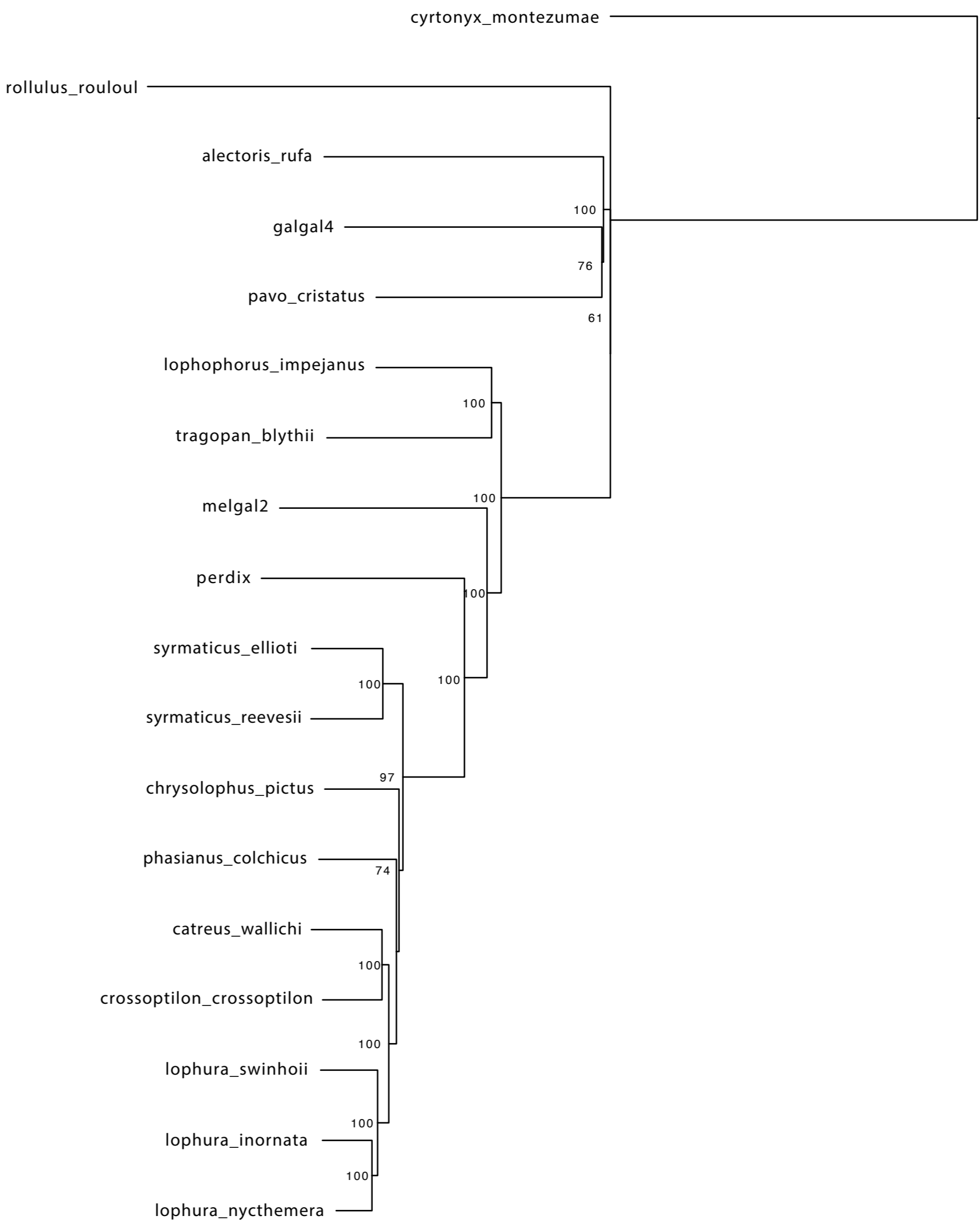

0.0050

Supplement: Figure S3 — Comparison of the tree topologies resulting from the Meiklejohn et al. and TIGER partitioning strategies. The tree from analysis with the same partitioning strategy as in the original study is shown on the left and the tree from the TIGER partitioned analysis is shown on the right. [file peerj-06-5498-s010.pdf]
